# Formulation of a glycolipid:lipopeptide mixture as biosurfactant-based dispersant and development of a low-cost glycolipid production process

**DOI:** 10.1038/s41598-022-20795-3

**Published:** 2022-09-29

**Authors:** Tipsuda Subsanguan, Nichakorn Khondee, Witchaya Rongsayamanont, Ekawan Luepromchai

**Affiliations:** 1 International Program in Hazardous Substance and Environmental Management, Graduate School, Chulalongkorn University, Bangkok, Thailand; 2 Research Program On Remediation Technologies for Petroleum Contamination, Center of Excellence On Hazardous Substance Management (HSM), Chulalongkorn University, Bangkok, Thailand; 3 Department of Natural Resources and Environment, Faculty of Agriculture Natural Resources and Environment, Naresuan University, Phitsanulok, Thailand; 4 Faculty of Environment and Resource Studies, Mahidol University, Nakhon Pathom, Thailand; 5 Department of Microbiology, Faculty of Science, Center of Excellence in Microbial Technology for Marine Pollution Treatment (MiTMaPT), Chulalongkorn University, Bangkok, Thailand

**Keywords:** Biotechnology, Microbiology, Environmental sciences, Engineering

## Abstract

Biosurfactant-based dispersants were formulated by mixing glycolipids from *Weissella cibaria* PN3 and lipopeptides from *Bacillus subtilis* GY19 to enhance the synergistic effect and thereby achieve hydrophilic-lipophilic balance. The proportions of each biosurfactant and dispersant-to-oil ratios (DORs) were varied to obtain the appropriated formulations. The most efficient glycolipid:lipopeptide mixtures (F1 and F2) had oil displacement activities of 81–88% for fuel and crude oils. The baffled flask test of these formulations showed 77–79% dispersion effectiveness at a DOR of 1:25. To reduce the cost of the dispersant, this study optimized the glycolipid production process by using immobilized cells in a stirred tank fermenter. Semicontinuous glycolipid production was carried out conveniently for 3 cycles. Moreover, food wastes, including waste coconut water and waste frying oil, were found to promote glycolipid production. Glycolipids from the optimized process and substrates had similar characteristics but 20–50% lower cost than those produced from basal medium with soybean oil in shaking flasks. The lowest cost dispersant formulation (F2*) contained 10 g/L waste-derived cell-bound glycolipid and 10 g/L lipopeptide and showed high dispersion efficiency with various oils. Therefore, this biosurfactant-based dispersant could be produced on a larger scale for further application.

## Introduction

Marine oil spill is a significant environmental issue especially in the coastal area. Several applications for oil spill remediation have been studied such as booms, skimmers, dispersants and biosurfactants^1,2^. Commercial chemical dispersants such as Corexit 9500, Superdispersant-25, Inipol 90 and Dasic Slickgone LTSW have been applied for combating crude and fuel oils^3^. However, they are composed of petroleum-based surfactants in organic solvents that have been found to be toxic or hazardous to marine organisms and ecosystems^4,5^. To produce green dispersants, several researchers formulated solvent-free biobased dispersants by mixing biosurfactants with other surfactants, such as mixtures of lipopeptide and sodium dihexyl sulfosuccinate (SDHS)^6^, lipopeptide and a palm oil-derived surfactant, Dehydol LS7TH^7^ and lactonic sophorolipid and ionic liquid surfactants^5^. Recently, a biosurfactant mixture of rhamnolipid (glycolipid) and exmulsins (a complex mainly composed of lipopeptides) has showed good dispersion performance compared with individual biosurfactants^8^. Thus, it is possible to formulate biosurfactant-based dispersants from microbial-based biosurfactant, which will be considered as green and sustainable products.

There are several methods to formulate dispersants containing surfactants, such as the hydrophilic-lipophilic deviation (HLD) concept^6,7^ and mass ratio^2,5,9^. The HLD concept is simple, but the characteristic curvature (Cc value) and molecular weight of the biosurfactants must be known, which is difficult to obtain due to the complexity of their molecular structures. Therefore, the easiest way to formulate biosurfactant-based dispersants is to mix each component by mass ratio or volume ratio. For example, Jin et al. reported that a mixture of soybean lecithin (L) and Tween-80 (T) at a ratio of 6:4 had the lowest interfacial tension at 0.075 mN/m^10^, while Zhu et al. mixed fish-based lipopeptide and dioctyl sulfosuccinate at a ratio of 8:2 and found 77% dispersion effectiveness with an ANS crude blend^2^. In this study, the mixtures of glycolipids from *Weissella cibaria* PN3 and lipopeptides from *Bacillus subtilis* GY19 at varying ratios were investigated glycolipids usually consist of a hydrophilic polysaccharide headgroup and one or more hydrophobic fatty acid tails^11^, whereas lipopeptides are composed of a hydrophobic moiety with a long fatty acid chain and some lipophilic amino acids in the head group^6,12^. Thus, their mixture could have a hydrophilic-lipophilic balance and promote oil dispersion.

Even though glycolipids and lipopeptides have several applications, they have some limitations due to their high production cost. Lipopeptides from *Bacillus subtilis* GY19 was economically produced according to Khondee et al.^13^, while the low-cost glycolipid production by *Weissella cibaria* PN3 has not been investigated. The bacterium was selected because it is a lactic acid bacterium (LAB), which has the ability to simultaneously produce extracellular and cell-bound glycolipids^14^. In addition, LAB have recently been recommended for industrial scale biosurfactant production because they are generally recognized as safe (GRAS)^15,16^. This study evaluated the glycolipid production process under a stirred tank fermenter and utilized food waste, including waste coconut water and waste frying oil, as substrates. Several food wastes have been applied for biosurfactant production, for example, waste canola oil for rhamnolipid^17^, waste cooking oil for glycolipid^18^, fruit residue waste for biosurfactant^19^, waste mango juice for glycolipoprotein^20^ and waste coconut water for mannosylerythritol A^21^.

In this study, the biosurfactant-based dispersants were formulated by varying the mass ratio of glycolipids and lipopeptides as well as the dispersant-to-oil ratio (DOR). The efficiency of either extracellular or cell-bound glycolipids to promote the activity of lipopeptides was evaluated with fuel and crude oils. To further reduce the cost of biosurfactant-based dispersant, the study focused on the development of a semicontinuous glycolipid production process by using immobilized bacterial inoculum and food wastes. The oil dispersion efficiency and cost of formulations containing waste-derived glycolipids were compared with the former formulation. The biosurfactant-based dispersant from waste-derived glycolipid will be suggested as an alternative green dispersant.

## Materials and methods

### Bacteria and chemicals

*Weissella cibaria* PN3 (MSCU 0840) and *Bacillus subtilis* GY19 (MSCU 0789) have been found to be effective glycolipid and lipopeptide producers, respectively^13,14^. These bacteria were deposited at the MSCU culture collection in the Department of Microbiology, Faculty of Science, Chulalongkorn University. Soybean oil and palm oil were purchased from Thai Vegetable Oil Public Company Limited, Bangkok, Thailand. Waste glycerol was supplied by Thai Oleochemicals Co., Ltd., Thailand. Waste coconut water and waste frying oil were purchased from local markets, Thailand. Waste coconut water was obtained from mature coconuts used for the production of coconut milk, while waste frying oil was a mixture of used soybean and palm oils. The chemical compositions of waste coconut water were 28.12 g/L total sugar, 0.056 g/L nitrogen, 5.37 g/L ash, 1.78 g/L potassium, 0.16 g/L calcium, 0.17 g/L sodium, 0.0086 g/L magnesium and 0.01 g/L iron. Crude oil (Bongkot light crude oil, BKC) and fuel oil (fuel c) were obtained from Thai Oil PCL and Bangchak Corporation PCL, respectively. All other chemicals were of analytical grade and purchased from Sigma–Aldrich Co., LLC. Two commercial dispersants, Slickgone NS type 2/3 (1–10% w/w anionic surfactant and > 50% kerosene) and Superdispersant-25 (1–10% dioctyl sulfosuccinate and 10–30% 2-butoxyethanol), were obtained from Thai Oil PCL company, Thailand, which selected these dispersants as a part of oil spill management plan in the country.

### Production and characterization of glycolipids and lipopeptides

Glycolipids were initially produced by *Weissella cibaria* PN3, which were immobilized on a porous carrier (Aquaporousgel, Nisshinbo Chemical Inc. Tokyo, Japan) and applied to shaking flasks for glycolipid production in batch mode^14^. The immobilized cells utilized basal medium supplemented with 2% (v/v) soybean oil for glycolipid production for 3 days^14^. Crude extracellular and cell-bound glycolipids were obtained by the solvent extraction process of cell-free broth and cell pellets of *Weissella cibaria* PN3, respectively. The glycolipid extraction process was followed by Subsanguan et al.^14^. Briefly, the culture broth was centrifuged at 8000 rpm for 10 min to separate between cell pellets and supernatant. The supernatant was initially extracted with 10% (v/v) hexane to remove residue oil whereas the cell pellets were washed with 0.85% (w/v) NaCl and centrifuged at 8000 rpm for 10 min. The cell-bound biosurfactant was recovered by resuspending the cell pellets in methanol for 1 h, then the sample was proceeded to the extraction process as used for extracellular biosurfactant in the supernatant after residual oil removal. Briefly, the pH of the sample was adjusted to 2.0 with 6 M HCl before adding an equal volume of a chloroform and methanol mixture (2:1 v/v). The solution was incubated in a rotary shaker at 200 rpm for 1 h. The organic solvent phase was separated from the water layer and evaporated by rotary evaporation. The viscous yellowish product was dissolved in methanol and filtered and the amount of crude biosurfactant was measured by weighing. Crude biosurfactant yield was calculated as g/L based on the volume of the production medium. For lipopeptides, chitosan-immobilized *Bacillus subtilis* GY19 was added to the productive medium supplemented with 10% (v/v) waste glycerol and 1.25% (v/v) palm oil and incubated for 5 days according to Khondee et al.^13^. Cell-free broth was obtained and used for crude lipopeptide extraction which was similar method to glycolipid extraction process. The stock biosurfactant solution was prepared by dissolving crude biosurfactant with phosphate buffer solution (PBS, pH 8.0). Although, our previous studies used freeze-dried powder of lipopeptides as a component in dispersant formulation^6,7^, this study used both lipopeptides and glycolipids as crude extracts so the biosurfactant solutions could be conveniently prepared according to their mass.

The surface tension (ST) of crude biosurfactant dissolved in phosphate buffer solution was measured using a digital tensiometer (Kruss, K10ST, Germany) at 25 °C using the plate method. The critical micelle concentration (CMC) was determined from a plot of surface tension versus biosurfactant concentrations. The properties of glycolipids and lipopeptides used for dispersant formulation were similar to those reported by Subsanguan et al.^14^ and Khondee et al.^13^, respectively. The surface tension and CMC value of extracellular glycolipids were 31.3 mN/m and 1.6 g/L, respectively, whereas cell-bound glycolipids had an ST of 32.6 mN/m and a CMC value of 3.2 g/L^14^. The values of surface tension and critical micelle concentration of the lipopeptide were 30.8 mN/m and 1.0 g/L, respectively^13^.

### Formulation of a biosurfactant-based dispersant

Biosurfactant-based dispersants were formulated by varying the mass ratio of each biosurfactant in PBS (pH 8.0). Initially, six formulations were prepared, which included three formulations of extracellular glycolipids and lipopeptides at mass ratios of 1:1, 1:2 and 1:4 and three formulations of cell-bound glycolipids and lipopeptides at the same mass ratios. The total concentration of each formulation was 20 g/L, which was higher than the CMC value of each biosurfactant. The oil dispersion efficiency of each formulation was investigated by an oil displacement test following Khondee et al.^13^. The DOR was initially varied at 1:15, 1:25 and 1:70 to cover the broad range of dispersant application. The synthetic seawater was prepared with NaCl at 3.4% followed by Rongsayamanont et al.^6^. The types of oil might affect the oil displacement activity of each formulation; thus, this study tested these biosurfactant mixtures with both crude and fuel oils. To confirm the efficiency of biosurfactant-based dispersants, the study repeated the experiment with DORs at 1:10, 1:15, 1:20 and 1:25 (Supplementary Table 1). The condition with the highest oil displacement activity was used to select the appropriated DOR and dispersant formulations including extracellular glycolipid:lipopeptide mixture (F1) and cell-bound glycolipid:lipopeptide mixture (F2). The oil displacement activity of individual biosurfactants at the appropriated DOR was later investigated to confirm the synergistic effect of the mixture. Finally, the dispersion effectiveness of biosurfactant-based dispersants for specific oils was investigated by a baffle flask test as in Nawavimarn et al.^7^. The dispersion effectiveness of the biosurfactant-based dispersants was compared with that of commercial dispersants. All samples were tested in triplicate. The cost of the biosurfactant-based dispersant was calculated based on the concentrations of glycolipids and lipopeptides in the formulation.

### Glycolipid production under a stirred tank fermenter

Stirred tank fermenter was used for preparation of immobilized *Weissella cibaria* PN3 and subsequent glycolipid production. The reactor allowed good interactions between oxygen, nutrients, and bacterial cells and would reduce the problem of substrate clogging over the carriers. For immobilization, 10% (v/v) bacterial inoculum (OD _600_ = 1.0) was added to a 2 L stirred tank fermenter containing 1% (w/v) porous carrier and 1.5 L LB medium and incubated for 2 days. The operation was controlled at 200 rpm agitation and at room temperature. The influence of aeration rates was investigated at 0.25, 0.5 and 1.5 vvm. The aeration rate plays an important role in biosurfactant production by microorganism especially lactic acid bacteria^22^.

The semicontinuous glycolipid production was initiated by replacing LB medium with basal medium containing 2% (v/v) soybean oil, while the operation was similar to the immobilization process. After 3 days, the culture broth was removed, where 50% of its volume was used for glycolipid extraction, and the remaining solution was returned to the fermenter for the next production cycle after the washing process (Supplementary Fig. 1). The previously produced glycolipids in culture broth would promote the solubilization of soybean oil in fresh productive medium; thus, this oil would be readily available for bacterial growth and glycolipid production. The immobilized bacteria were washed with phosphate buffered saline (PBS, pH 8.0) after each production cycle because the immobilizing carriers are usually clogged by residual soybean oil and some metabolites^14^. The production and washing process was repeated for a total of 3 cycles. The culture broth was centrifuged to separate cell pellets for cell-bound glycolipid extraction, while the cell-free broth was used for extracellular glycolipid extraction^14^. The concentration of each glycolipid type was based on the volume of productive medium used for each cycle. The characteristics of immobilized bacteria were recorded using a scanning electron microscope and energy dispersive X-ray spectrometer (SEM–EDS, IT500HR).

### Utilization of food wastes as substrates for glycolipid production

Basal medium containing 2% (v/v) waste frying oil and waste coconut water supplemented with 2% (v/v) waste frying oil were investigated as a low-cost productive medium. Waste frying oil from cooking and waste coconut water from coconut milk production are selected since they are easy to find year-round and are inexpensive. The costs of extracellular and cell-bound glycolipids from different productive media were calculated based on the total glycolipid concentration from three production cycles and the relevant costs of supplies and production processes, including chemicals, carbon sources, fermentation, centrifugation and evaporation during the recovery process, and solvents for biosurfactant extraction.

The surface tension and CMC value of crude glycolipids derived from food wastes were determined followed Subsanguan et al.^14^ to understand whether the impurities in waste substrates affected their surface properties. In addition, waste-derived crude glycolipids were mixed with lipopeptides to formulate lower-cost biosurfactant-based dispersants. The dispersion efficiency of formulations containing waste-derived glycolipids and lipopeptides was examined and compared to that of the former formulations. For structure characterization, the crude glycolipids were partially purified by ultrafiltration techniques as described by Subsanguan et al.^14^. The functional groups of the purified biosurfactants were determined by Fourier transform infrared (FTIR) spectroscopy in ATR mode (Spectrum, GX, Perkin Elmer) at wavenumbers ranging from 4000 to 400 cm^−1^ and a resolution of 0.3 cm^−1^.

## Results and discussion

### Formulation of biosurfactant-based dispersants

**Figure 1:**
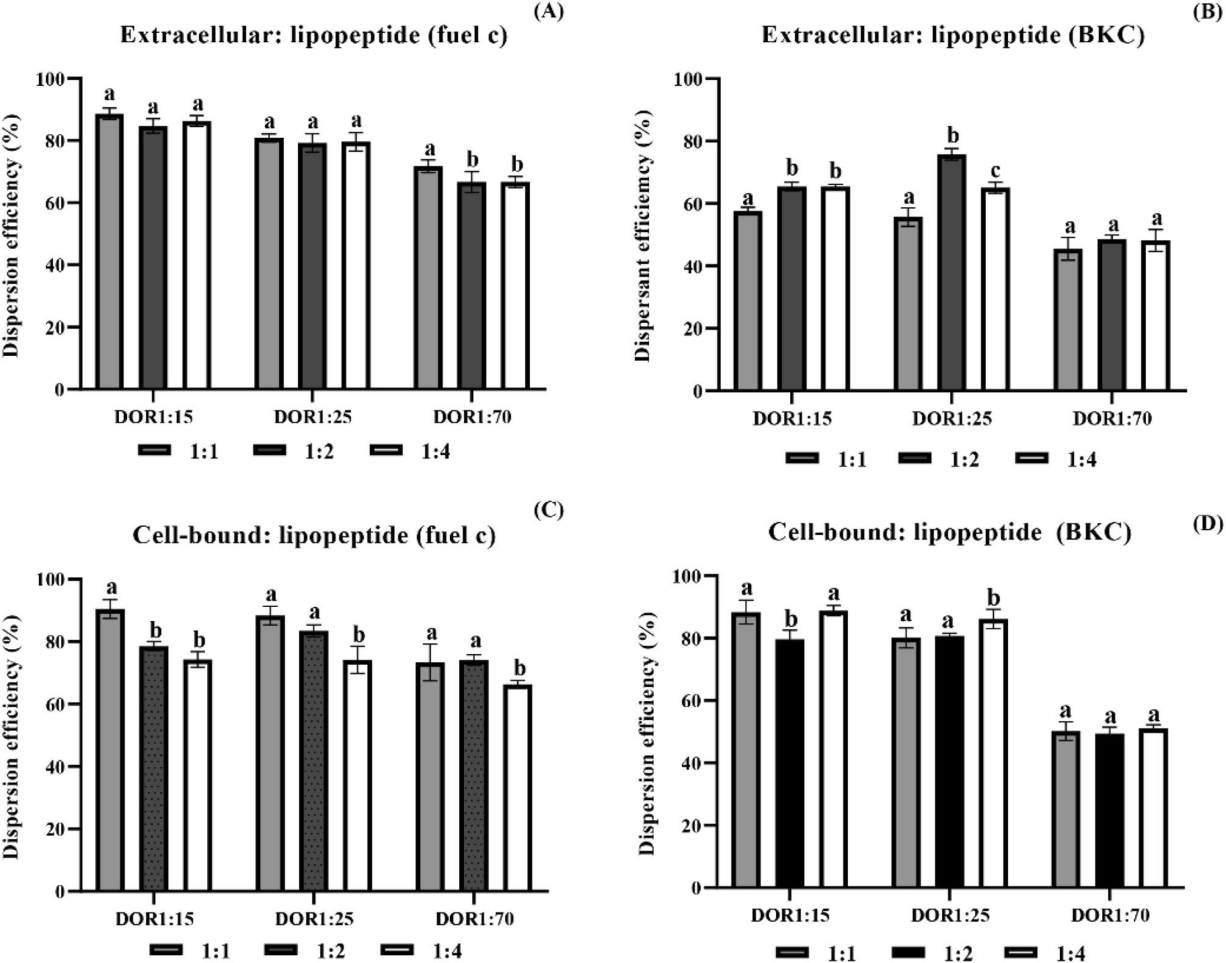
Oil displacement activity of extracellular glycolipid:lipopeptide mixtures (**A**,**B**) and cell-bound glycolipid:lipopeptide mixtures (**C**,**D**) at mass ratios of 1:1, 1:2 and 1:4. The tested oils were fuel oil (**A**,**C**) and BKC crude oil (**B**,**D**). Error bars represent the mean ± standard deviation (n = 3). Two-way ANOVA: Tukey’s multiple comparison test was used for statistical analysis. Different letters represent statistically significant differences (*p* < 0.05).

The mixtures of extracellular glycolipid with lipopeptide and cell-bound glycolipid with lipopeptide were effective for oil dispersion, of which the oil displacement activity ranged from 66 to 90% for fuel oil and 45–88% for crude oil in the initial experiment (Fig. 1). When compared between different DOR values, all biosurfactant mixtures showed low oil displacement activity at a DOR of 1:70. When compared between different ratios of glycolipid and lipopeptides, the results showed varying results. The formulations with increasing hydrophobicity (i.e., more lipopeptides) were initially expected to have high oil displacement activity. However, the efficiency of biosurfactant mixtures was not significantly different (*p* > 0.05), especially extracellular glycolipid and lipopeptide mixtures for fuel oil and cell-bound glycolipid and lipopeptide mixtures for crude oil (Fig. 1A,D). For crude oil, the extracellular glycolipid and lipopeptide mixture at a mass ratio of 1:2 and DOR of 1:25 had the highest oil displacement activity (Fig. 1B). On the other hand, the formulation containing cell-bound glycolipid and lipopeptide at a mass ratio of 1:1 and DOR of 1:15 and 1:25 showed the highest oil displacement activity for fuel oil (Fig. 1C). The results suggested that the interaction between biosurfactants and oils was complex, which was due to the varying characteristics of biosurfactants and oils.

When more DOR values were investigated, the appropriated biosurfactant-based dispersants were obtained (Supplementary Table 1). The most efficient formulations were extracellular glycolipid:lipopeptide mixture at 1:2 mass ratio (F1) and cell-bound glycolipid: lipopeptide mixture at 1:1 mass ratio (F2). At the appropriated DOR, all formulations exhibited higher oil displacement activity (81–88%) than a single biosurfactant (55–77%) for both fuel and crude oils (Table 1). It was observed that the mixing of glycolipids with lipopeptides had a synergistic effect on oil displacement activity. The extracellular glycolipid from *Weissella cibaria* PN3 is more hydrophilic than its cell-bound glycolipid^14^. Consequently, the formulations containing extracellular glycolipids required more lipopeptide mass to achieve hydrophilic-lipophilic balance. Both selected formulations corresponded with the hypothesis. The formulations of solvent-free oil dispersants containing lipopeptides usually require another hydrophilic chemical surfactant, such as SDHS (an anionic synthetic surfactant)^6^ or dehydol LS7TH (a nonionic oleic surfactant)^7^. The use of glycolipids instead of other synthetic surfactants qualifies these formulations as green dispersants for oil spill remediation. The effect of other environmental factors, such as temperature and salinity, on the dispersion effectiveness of the dispersants should be determined further.

**Table 1:** Comparison of oil displacement activities of biosurfactant-based dispersants and individual biosurfactants under various conditions.

Tested oil	Formulation	Condition		Oil displacement activity (%)
		BSF ratio^a^	DOR	
Fuel oil	F1 (Extracellular glycolipid: Lipopeptide)	1.0:2.0	1:10	85.49 ± 1.80
	F2 (Cell-bound glycolipid: Lipopeptide)	1.0:1.0	1:20	88.24 ± 2.04
	Extracellular glycolipid	1.0	1:10	73.75 ± 1.25
	Cell-bound glycolipid	1.0	1:20	62.50 ± 2.50
	Lipopeptide	1.0	1:10	75.42 ± 3.15
	Lipopeptide	1.0	1:20	56.25 ± 3.75
BKC crude oil	F1 (Extracellular glycolipid: Lipopeptide)	1.0:2.0	1:20	81.18 ± 1.18
	F2 (Cell-bound glycolipid: Lipopeptide)	1.0:1.0	1:20	85.88 ± 1.18
	Extracellular glycolipid	1.0	1:20	77.92 ± 0.72
	Cell-bound glycolipid	1.0	1:20	55.42 ± 1.44
	Lipopeptide	1.0	1:20	74.58 ± 2.60

^a^BSF ratio is the mass ratio of extracellular/cell-bound glycolipid to lipopeptide.

### Dispersion effectiveness of biosurfactant-based dispersants and cost analysis

**Figure 2:**
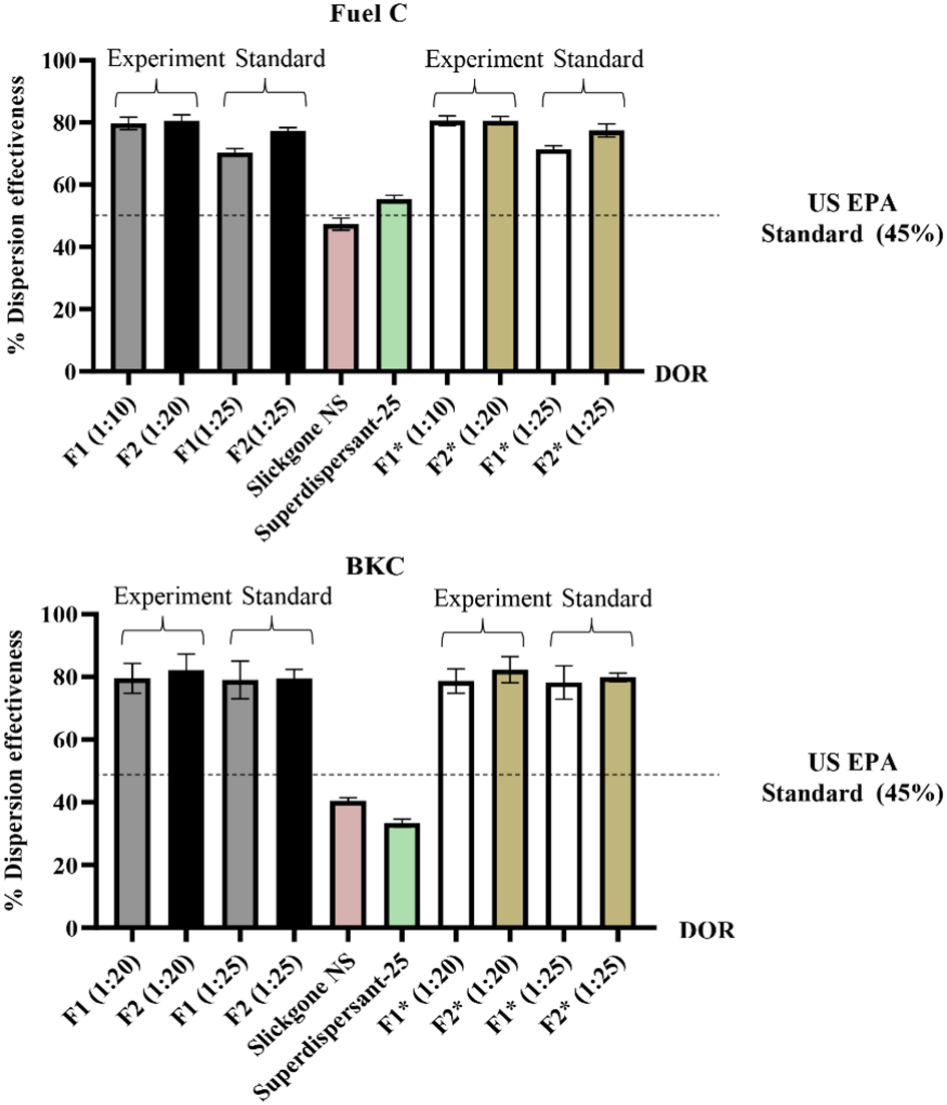
Dispersion effectiveness (%) of biosurfactant-based dispersants and commercial dispersants for fuel oil and BKC crude oil. F1 is an extracellular glycolipid:lipopeptide mixture, while F2 is a cell-bound glycolipid:lipopeptide mixture. The star indicates that glycolipids in the formulation were obtained from waste coconut water supplemented with waste frying oil. *Experiment is the DOR obtained from the condition in Table 1, while standard is the recommended DOR (1:25) from the standard method for baffled flask test.

The selected formulations, F1 and F2 were further investigated to confirm the dispersion effectiveness by a modified baffled flask test. All biosurfactant-based dispersants exhibited high dispersion effectiveness for both fuel and crude oils. The F2 formulation had the highest dispersion effectiveness at 81% and 83% when applied at the appropriated DOR to fuel and crude oils, respectively (Fig. 2). This formulation contained 10 g/L cell-bound glycolipids and 10 g/L lipopeptides (Supplementary Table 2). To compare the dispersion efficiency with other dispersants, the tested formulations were investigated at a DOR of 1:25. Formulation F2 had slightly lower dispersion effectiveness at a DOR of 1:25, but they still showed the highest dispersion effectiveness of 77% and 79% with fuel and crude oils, respectively (Fig. 2). All formulated biosurfactant-based dispersants met the US EPA standard for oil spill dispersants, for which the dispersion effectiveness values were greater than 45%.^23^ In addition, these formulations had higher dispersion effectiveness than the commercial dispersants Slickgone NS and Superdispersant-25 (Fig. 2).

The biosurfactant-based dispersants dispersed crude oil to a slightly greater extent than fuel oil (Fig. 2). This is because crude oil has low density and viscosity and is composed of only saturated hydrocarbons, whereas the composition of fuel oil is saturated and aromatic hydrocarbons, resin and asphaltene^6,7^. The efficiency of these biosurfactant-based dispersants was comparable with other studies. For example, a mixture of rhamnolipid and exmulsins has 87% oil removal performance for ALC crude oil^8^, a formulation of lactonic sophorolipid with choline laurate shows 88% dispersion effectiveness with Tapis light crude oil^9^ and a mixture of lipopeptide and dioctyl sulfosuccinate sodium (DOSS) exhibits 77% dispersion effectiveness with an ANS crude blend^2^.

The cost of biosurfactant-based dispersants was dependent on the cost of glycolipids and lipopeptides. In this study, the synergistic effect of glycolipid and lipopeptide molecules allowed the use of lower lipopeptide concentrations than those in the other lipopeptide-containing dispersants. The concentration of lipopeptides in the F2 formulation was 1.0% w/v (Supplementary Table 2), while other dispersants usually contain much higher lipopeptide concentrations, for example, 7.0% (w/v) in the lipopeptide-SDHS formulation^6^ and 6.6% (w/v) in the lipopeptide-dehydol LS7TH formulation^7^. Decreasing the lipopeptide concentration reduced the cost of these biosurfactant-based dispersants. However, the costs of biosurfactant-based dispersants were significantly higher than those of commercial dispersants, e.g., COREXIT 9500 and Slickgone NS (Supplementary Table 2). This was due to the high biosurfactant production cost and low biosurfactant yield, especially for glycolipids. Thus, the following experiment focused on optimization of the glycolipid production process.

### Glycolipid production by immobilized bacteria in a stirred tank fermenter

**Figure 3:**
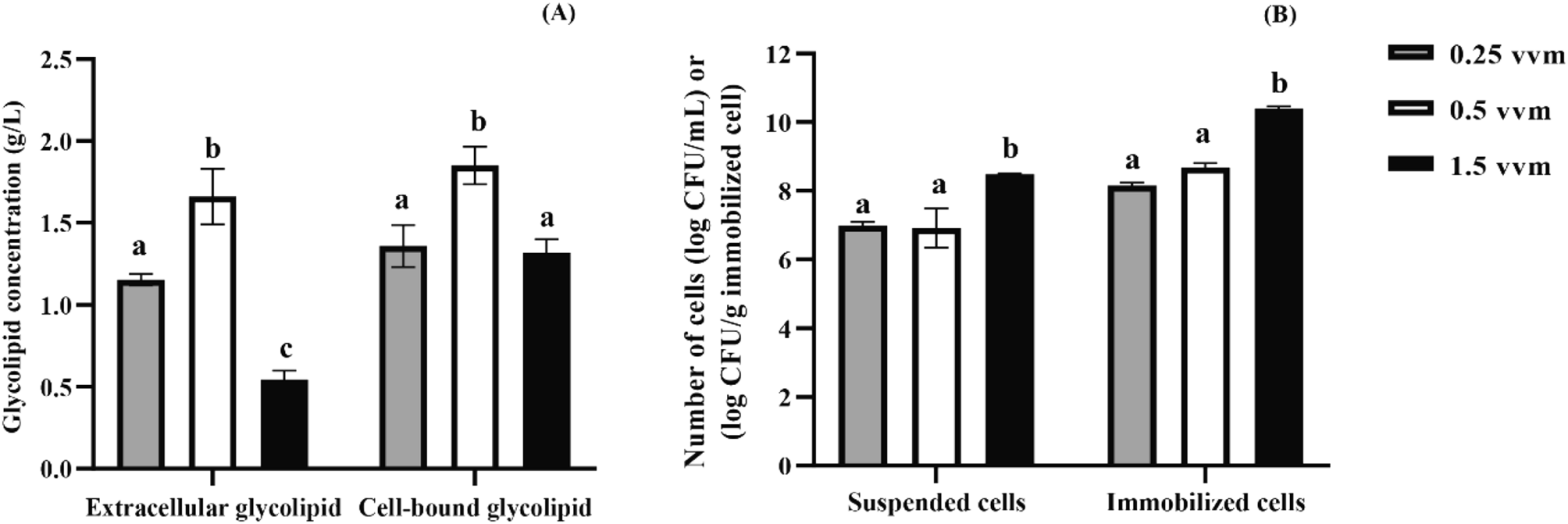
Crude glycolipid concentrations (**A**) and bacterial cell numbers (**B**) in a stirred tank fermenter with different aeration rates. The glycolipid concentration was based on the volume of productive medium. Error bars represent the standard deviation of the mean (n = 3). Two-way ANOVA: Tukey’s multiple comparison test was used for statistical analysis. Different letters represent statistically significant differences (*p* < 0.05).

Immobilized *Weissella cibaria* PN3 was able to produce extracellular and cell-bound glycolipids simultaneously under a stirred tank fermenter. The maximum yield of extracellular and cell-bound glycolipid was 1.66 and 1.85 g/L at 0.5 vvm aeration rate, respectively, which was significantly different (*p* < 0.05) from other aeration rates (Fig. 3A). The concentration of extracellular glycolipids at 0.5 vvm aeration was three times higher than that of the aeration rate at 1.5 vvm, whereas the concentration of cell-bound glycolipids was increased at one time. Comparing the number of bacterial cells, the number of immobilized cells at 0.5 vvm (8.68 log CFU/g immobilized cells) was significantly lower than that at 1.5 vvm (10.46 log CFU/g immobilized cells) (Fig. 3B). Similarly, Gudiña et al. reported that biosurfactant yield from Lactobacillus strains was low at high aeration rate, while the biomass concentrations were high^22^. The high agitation can promote excessive foam, resulting in a decreased biosurfactant yield and difficulty in process control^24^. In this study, the stirred tank fermenter containing immobilized *Weissella cibaria* PN3 was operated at low aeration rate of 0.5 vvm without excessive foaming (Supplementary Fig. 2A). The results indicated that the process was suitable for glycolipid production.

When basal medium and soybean oil were used as substrates, the immobilized *Weissella cibaria* PN3 produced 1.59 g/L extracellular glycolipids and 1.77 g/L cell-bound glycolipids after the 1st production cycle (Table 2). The glycolipid concentrations gradually decreased in the 2nd and 3rd production cycles. The total concentrations of obtained glycolipids were comparable to those produced in shaking flasks with similar substrates^14^. Comparing the number of bacterial cells in each cycle, the number of suspended and immobilized cells was almost constant over 3 production cycles (Table 2). However, the SEM analysis showed that washed immobilizing carrier after the 3rd cycle had some metabolites or biofilm covering the surface more than those in the 1st cycle (Supplementary Fig. 3). The results corresponded with the decreasing trend of glycolipids and suggested mass transfer limitation after several glycolipid production cycles. Nonetheless, the stirred tank fermenter allowed the large-scale production of glycolipids. The semicontinuous glycolipid production also decreased the volume of productive medium and production cost. Other parameters influencing glycolipid production, such as dissolved oxygen, substrate types and concentrations, pH and temperature, should be further investigated to increase biosurfactant yields.

**Table 2:** Semicontinuous production of glycolipids using different productive media under a stirred tank fermenter.

Productive medium and condition	Cycle	Extracellular glycolipid (g/L)	Cell-bound glycolipid (g/L)	Suspended cells (log CFU/mL)	Immobilized cells (log CFU/g immobilized cells)
Basal medium with soybean oil in shaking flasks*	1	1.42 ± 0.15^a^	1.69 ± 0.23^a^	8.23 ± 0.17^a^	8.70 ± 0.57^a^
	2	1.29 ± 0.34^a^	1.39 ± 0.07^a^	8.17 ± 0.05^a^	8.87 ± 0.32^a^
	3	1.04 ± 0.15^a^	1.28 ± 0.28^a^	8.19 ± 0.13^a^	8.35 ± 0.55^a^
Basal medium with soybean oil in stirred tank fermenter	1	1.59 ± 0.05^a^	1.77 ± 0.13^a^	8.61 ± 0.07^a^	8.56 ± 0.04^a^
	2	1.29 ± 0.16^b^	1.51 ± 0.11^a^	8.57 ± 0.07^a^	8.60 ± 0.03^a^
	3	1.14 ± 0.15^b^	1.28 ± 0.06^b^	8.58 ± 0.15^a^	8.51 ± 0.02^a^
Basal medium with waste frying oil in stirred tank fermenter	1	1.05 ± 0.15^a^	1.47 ± 0.12^a^	8.04 ± 0.16^a^	8.56 ± 0.06^a^
	2	0.86 ± 0.03^a^	1.24 ± 0.02^b^	7.39 ± 0.08^b^	8.85 ± 0.49^a^
	3	0.64 ± 0.10^b^	1.19 ± 0.09^b^	7.29 ± 0.11^b^	7.98 ± 0.12^b^
Waste coconut water with waste frying oil in stirred tank fermenter	1	1.59 ± 0.15^a^	2.68 ± 0.32^a^	9.38 ± 0.08^a^	10.38 ± 0.07^a^
	2	1.44 ± 0.11^a^	2.74 ± 0.11^a^	9.22 ± 0.10^b^	10.46 ± 0.05^a^
	3	1.48 ± 0.19^a^	2.67 ± 0.15^a^	9.09 ± 0.09^b^	10.41 ± 0.03^a^

The glycolipid concentrations were based on the volume of productive medium, while the numbers of suspended and immobilized cells were determined at the end of the production cycle.

Data are presented as the mean ± standard deviation (n = 3). Two-way ANOVA: Tukey’s multiple comparison test was used for statistical analysis. Different letters represent statistically significant differences (*p* < 0.05) in different production cycles within the same productive medium and condition.

*The data were obtained from^14^.

### Utilization of waste frying oil and waste coconut water as substrates for glycolipid production

Waste frying oil was initially examined as a low-cost glycolipid substrate for semicontinuous glycolipid production. The concentrations of extracellular and cell-bound glycolipids from waste frying oil ranged from 0.64–1.05 g/L and 1.19–1.47 g/L, respectively, which were lower than when soybean oil was used as substrate (Table 2). The glycolipid concentrations in the 3rd cycle were significantly decreased (*p* < 0.05), which was correlated with an approximately 1 order of magnitude decrease in immobilized and suspended cells in the presence of waste frying oil (Table 2). This was probably due to the changes in the properties and structure of waste frying oil through heating from cooking. In this study, waste frying oil was a mixed vegetable oil that was used several times. It had high viscosity in the stirred tank fermenter (Supplementary Fig. 2B,C). Several researchers have studied biosurfactant production using oily waste. Hisham et al. reported that *Bacillus* sp. HIP3 produced the maximum biosurfactant at 5.35 g/L using 2% v/v of used cooking oil as substrate^25^. Pérez-Armendáriz reported that *Pseudomonas aeruginosa* produced rhamnolipid at 3.2 g/L with canola oil and 3.6 g/L with waste canola oil^17^. In addition, oily substrates can be applied in combination with hydrophilic substrates to enhance biosurfactant production, for example, soybean cooking oil and corn steep liquor for producing lipoprotein biosurfactants^26^; and palm oil effluent and crude glycerol for producing lipopeptides^27^. It might be possible to change the type of waste frying oil to promote glycolipid production in future studies.

To further reduce the cost of glycolipid production, the basal medium was replaced with waste coconut water, which could provide hydrophilic substrates for the bacteria. The results showed that immobilized cells in a stirred tank fermenter containing waste coconut water and waste frying oil produced 1.59 g/L extracellular glycolipid and 2.68 g/L cell-bound glycolipids after the 1st production cycle, which were higher than those from basal medium supplemented with waste frying oil (Table 2). The increasing concentrations of extracellular and cell-bound glycolipids corresponded with increasing bacterial growth. When waste coconut water and waste frying oil were used as the productive medium, the numbers of suspended cells and immobilized cells from each production cycle ranged from 9.09 to 9.38 log CFU/mL and 10.38–10.41 log CFU/g immobilized cells, respectively (Table 2). In addition, SEM analysis showed more bacterial cells attached to the immobilizing carriers in waste coconut water with waste frying oil (Supplementary Fig. 3E–H) than in basal medium with soybean oil (Supplementary Fig. 3A–D). Almost constant glycolipid concentrations were obtained during the 1st to 3rd production cycles (Table 2); therefore, it is possible to use immobilized cells in waste coconut water with waste frying oil for more than 3 cycles to increase the glycolipid yield. Other studies also reported the application of waste coconut water to promote biosurfactant production^21,28,29^.

The maximum biosurfactant production is usually found when the ratio of carbon and nitrogen sources enter nitrogen-limiting conditions^29^. When comparing the C:N ratio of each productive medium, waste coconut water supplemented with waste frying oil provided the highest C:N ratio of 824.64 (Table 3). Similarly, Prieto et al. reported that *P. aeruginosa* LBM10 produced a biosurfactant yield of 1.42 g/L in a nitrogen limiting condition (C/N ratio of 100), whereas the biosurfactant yield was decreased to 0.94 g/L at a C/N ratio of 22^30^. By using waste coconut water and waste frying oil, the nitrogen limiting condition was reached, and glycolipid production was increased. When comparing the cost, waste coconut water supplemented with waste frying oil had the lowest cost (Table 3), while it provided the highest concentrations of both extracellular and cell-bound glycolipids (Table 2). Consequently, glycolipids from the optimized process and substrates had 20–50% lower cost than those produced from basal medium with soybean oil in shaking flasks (Table 3). It is possible to apply waste coconut water with waste frying oil for scale-up glycolipid production.

**Table 3:** Cost of productive media and their C:N ratio.

Productive medium*	C:N ratio	Medium cost (USD/L)	Glycolipid cost (USD/g)		Glycolipid properties			
			Extracellular	Cell-bound	Surface tension (mN/m)		CMC (g/L)	
					Extracellular	Cell-bound	Extracellular	Cell-bound
Basal medium with soybean oil**	2.3	0.87	2.72	2.34	31.3	32.6	1.6	3.2
Basal medium with soybean oil	2.3	0.86	2.54	2.24	33.8	34.4	2.0	3.7
Basal medium with waste frying oil	2.3	0.85	4.00	2.61	43.5	40.8	3.0	3.6
Waste coconut water with waste frying oil	824.6	0.11	2.10	1.17	36.2	39.1	2.9	3.4

The cost of glycolipids and their properties are also shown.

*The costs of productive media were calculated from the price of each component purchased at local markets, while glycolipid costs were based on the glycolipid concentrations obtained from each medium (Table 2). Basal medium price 0.72 USD/L Reference: M&P IMPEX LTD, Thailand.

The soybean oil price is 1.57 USD/L, which was purchased from Thai Vegetable Oil Public Company Limited. The waste coconut water price is 0.10 USD/L, which was purchased from the Khlong Toei market, Bangkok, Thailand, and the price of waste frying oil was 0.49 USD/L, which was purchased from Baan Lad, Phetchaburi Province, Thailand. The C:N ratio of each productive medium was determined from the components of basal medium, waste coconut water, soybean oil and waste frying oil.

**The data were obtained from Subsanguan et al.^14^, and glycolipid production was carried out in shaking flasks.

### Application of glycolipids derived from waste as components in biosurfactant-based dispersants

Before application, the crude extracellular and cell-bound glycolipids from several production cycles were mixed and determined their surface activity (Supplementary Fig. 4). The highest surface tension values of glycolipids from semicontinuous production were derived from: waste frying oil alone, then waste coconut water and waste frying oil, and finally basal medium and soybean oil; the highest CMC values were derived from the substrates in the opposite order (Table 3). The results suggested that different glycolipid production processes and substrates could affect the biosurfactant’s surface activity. Waste frying oil contained some impurities, which could remain in the crude glycolipids and reduce their surface activities. The impurities were clearly seen when comparing the FTIR spectra of glycolipids derived from waste coconut water and waste frying oil and those derived from basal medium and soybean oil (Supplementary Fig. 5). To achieve a consistent level of surface activity, Cai et al. suggested using the CMC of biosurfactants for the selection of the appropriate biosurfactant dose in products^8^.

The application of crude glycolipids derived from waste coconut water and waste frying oil as a component in biosurfactant-based dispersants was investigated by mixing with lipopeptides. The formulations were defined as F1* and F2*, which had similar compositions to their respective formulations, F1 and F2 (Table 1). These formulations had good dispersion effectiveness (> 75%) for both fuel and crude oils at a DOR of 1:25. The results indicated that impurities in waste-derived glycolipids did not affect the overall dispersant’s efficiency. The dispersant costs were calculated from the cost of biosurfactants. The highest efficiency formulation, F2* had the lowest cost which was at the same level as Corexit 9500A (Supplementary Table 2). It was found that the cost of biosurfactant was mainly due to the solvent extraction process (Supplementary Fig. 6), of which a high volume of solvent was used in this study. To avoid the use of solvent, biosurfactants might be concentrated from culture broth using freeze drying technique and applied in the dispersant formulation. For example, the freeze-dried lipopeptide powder is used in a dispersant composed of lipopeptide and SDHS^6^.

The efficiency of F2* formulation was later challenged with different petroleum oils. The oil displacement activity of the F2* formulation at a DOR of 1:25 was 79% for fuel c, 77% for ARL crude oil, 83% for engine oil and 86% for gasoline (Supplementary Fig. 7). The dispersion effectiveness of this formulation was in the same range with other green dispersants by^2,5,9^. The high dispersion efficiency of the glycolipid and lipopeptide mixture could be due to the large micelle formation. The major lipopeptide in *Bacillus subtilis* GY 19 is surfactin, which consists of a fatty acid chain in the hydrophobic part and several amino acids in the hydrophilic group^6^. On the other hand, glycolipids are composed of a long-chain fatty acid and a small part of hydrophilic groups, such as di-rhamnolipid^31^ and lactonic sophorolipid^32^. This study produced lipopeptides from palm oil, which had palmitic acid (C16-0) as the major fatty acid. The molecules of lipopeptides with a C16 fatty acid tail would align between the glycolipid molecules with long-chain fatty acid, thus allowing the formation of a large hydrophobic center to accommodate the high volume of oil solubilization. The large hydrophilic head of the lipopeptide also reduced the interfacial tension between oil and water. Therefore, the F2* formulation could be applied as a universal green dispersant for combating oil spills in the environment.

## Conclusion

Biosurfactant-based dispersants were simply formulated by mixing crude glycolipids and lipopeptides, in phosphate buffer solution. The synergistic effect of these two biosurfactants allowed the hydrophilic-lipophilic balance and large micelle formation. This approach could be applied to the formulation of other biosurfactant-based products. The cost of dispersants was significantly reduced by using glycolipids from the semicontinuous production process and the utilization of waste coconut water with waste frying oil as a productive medium. The F2* formulation containing 10 g/L waste-derived cell-bound glycolipids and 10 g/L lipopeptides had the lowest cost and could be used to disperse various oils. In summary, this study emphasizes the potential of formulating biosurfactant-based dispersants and the benefit of using food wastes as biosurfactant substrates. It is thus possible to produce biosurfactant-based dispersants on a larger scale for further application.

## Supplementary Information

Supplementary Information.

## Data Availability

All data generated or analysed during this study are included in this published article [and its supplementary information files].
