# Supplementary material for: Formulation of a glycolipid:lipopeptide mixture as biosurfactant-based dispersant and development of a low-cost glycolipid production process
Source: Sci Rep. 2022 Sep 29;12:16353. doi: 10.1038/s41598-022-20795-3 (PMC9522864; doi:10.1038/s41598-022-20795-3)
Supplement: Supplementary file 1 — Supplementary Information. [file 41598_2022_20795_MOESM1_ESM.docx]

**Supplementary Materia****l**

**Formulation of a glycolipid:lipopeptide mixture as biosurfactant-based dispersant and development of a low-cost glycolipid production process**

**1. Formulation of biosurfactant-based dispersant**

**Supplementary Table 1. Oil displacement activity of glycolipid:lipopeptide mixtures when applied to fuel and crude oils at varying biosurfactant ratio and DOR.**

| Run no. | Extracellular glycolipid:lipopeptide mixture | | | |
| --- | --- | --- | --- | --- |
|  | **Biosurfactant ratio** | **DOR** | **Oil displacement activity (%)** | |
|  |  |  | **Fuel oil** | **BKC crude oil** |
| 1 | 1:4 | 1:25 | 79.22±1.80 | 67.84±2.96 |
| 2 | 1:4 | 1:20 | 80.31±1.36 | 69.80±1.36 |
| 3 | 1.4 | 1.15 | 84.31±3.78 | 64.71±1.18 |
| 4 | 1.4 | 1:10 | 82.35±2.35 | 71.77±2.04 |
| 5 | 1:2 | 1:25 | 79.61±1.80 | 72.15±3.78 |
| 6 | 1:2 | 1:20 | 80.00±1.18 | 81.18±1.18 |
| 7 | 1:2 | 1:15 | 84.31±2.72 | 65.10±2.45 |
| 8 | 1:2 | 1:10 | 85.49±1.80 | 73.33±1.80 |
| 9 | 1:1 | 1:25 | 80.78±0.68 | 54.12±2.35 |
| 10 | 1:1 | 1:20 | 81.96±1.80 | 56.83±1.80 |
| 11 | 1:1 | 1:15 | 84.31±1.80 | 57.65±1.18 |
| 12 | 1:1 | 1:10 | 82.35±1.18 | 59.61±2.96 |

**Supplementary Table 1 (cont.). Oil displacement activity of glycolipid:lipopeptide mixtures when applied to fuel and crude oils at varying biosurfactant ratio and DOR.**

| Run no. | Cell-bound glycolipid:lipopeptide mixture | | | |
| --- | --- | --- | --- | --- |
|  | **Biosurfactant ratio** | **DOR** | **Oil displacement activity (%)** | |
|  |  |  | **Fuel oil** | **BKC crude oil** |
| 1 | 1:4 | 1:25 | 74.51±1.80 | 83.14±1.36 |
| 2 | 1:4 | 1:20 | 75.29±1.18 | 83.92±2.45 |
| 3 | 1.4 | 1.15 | 74.12±3.11 | 83.53±1.18 |
| 4 | 1.4 | 1:10 | 76.47±1.18 | 84.31±1.79 |
| 5 | 1:2 | 1:25 | 81.96±0.68 | 81.60±0.67 |
| 6 | 1:2 | 1:20 | 82.75±1.80 | 83.53±1.18 |
| 7 | 1:2 | 1:15 | 80.00±2.04 | 82.35±2.35 |
| 8 | 1:2 | 1:10 | 81.96±2.45 | 83.14±2.45 |
| 9 | 1:1 | 1:25 | 84.31±1.80 | 84.31±1.34 |
| 10 | 1:1 | 1:20 | 88.24±2.04 | 85.88±1.18 |
| 11 | 1:1 | 1:15 | 85.88±3.53 | 83.17±1.36 |
| 12 | 1:1 | 1:10 | 85.10±2.45 | 84.31±0.68 |

**Supplementary Table 2.** **Cost of biosurfactant-based dispersants and commercial dispersants**

| Formulation^a^ | Glycolipid productive medium | Biosurfactant composition (g/L) | Cost (USD/kg)^b^ |
| --- | --- | --- | --- |
| F1 | Basal medium with  Soybean oil | Extracellular glycolipid (6.67): Lipopeptide (13.33) | 21.84 |
| F2 |  | Cell-bound glycolipid (10.00): Lipopeptide (10.00) | 25.88 |
| F1* | Waste coconut water with  Waste frying oil | Extracellular glycolipid (6.67): Lipopeptide (13.33) | 18.96 |
| F2* |  | Cell-bound glycolipid (10.00): Lipopeptide (10.00) | 15.39 |
| Corexit 9500A |  | - | 12.97 |
| Slickgone NS |  | - | 3.52 |

^a^The F1* and F2*formulations contained waste derived glycolipids.

^b^The cost of glycolipid from different productive medium was from Table 3, while the lipopeptide cost was 0.4 USD/g based on the production process reported by Khondee et al. (2015).

Source: The price of the dispersant Corexit 9500 was obtained from Clean Caribbean & Americas
(CCA) and Oil Spill Response, Limited, while the price of Slickgone was obtained from Oil Spill Response Limited.

**2. Glycolipid production under a stirred tank fermenter**

Adding 100% productive medium and carbon source

*Weissella cibaria* PN3 on MRS agar

Inoculating into LB medium in a stirred tank fermenter with 1% (w/v) aquaporousgel

1^st^ glycolipid production cycle

2^nd^ glycolipid production cycle

Washing with PBS (pH 8.0)

Immobilized *Weissella cibaria* PN3

Washing with PBS (pH 8.0)

3^rd^ glycolipid production cycle

Removal of culture medium

Adding 100% productive medium and carbon source

Incubating at 37 °C for 3 days

1^st^ glycolipid production cycle

Incubating at RT, 2 days

Removal of LB medium

Incubating at RT, 3 days

50% of culture medium

Removal of culture medium

Incubating at RT, 10 mins

50% of

culture medium

50% of culture medium

Glycolipid extraction from cell pellets

and supernatant

Removal of culture medium

Incubating at RT, 3 days

Adding 100% productive medium and carbon source

Incubating at RT, 10 mins

50% of culture medium

Incubating at RT, 3 days

100% of culture medium

**Supplementary Figure 1.** Glycolipid semicontinuous production process using a stirred tank fermenter containing immobilized-*Weissella cibaria*.


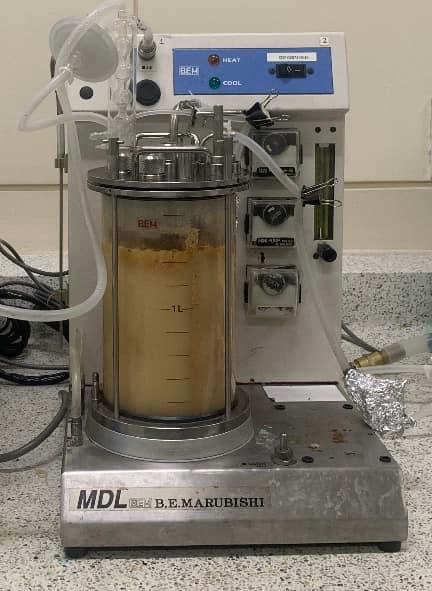


**(C)**


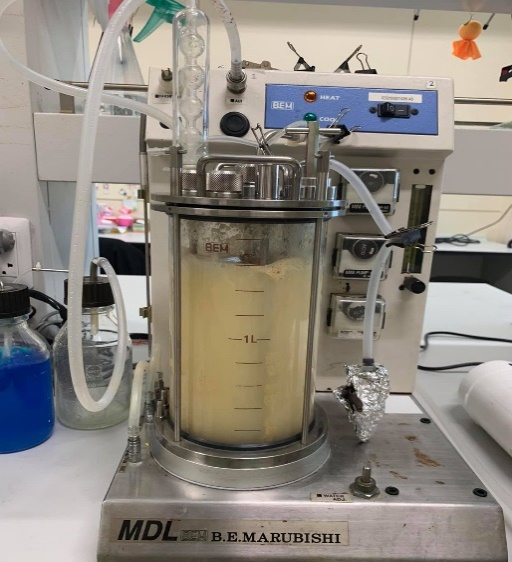


**(B)**


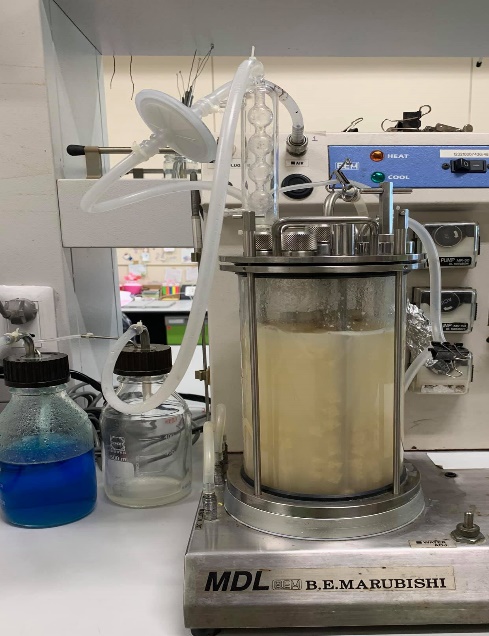


**(A)**

**Supplementary Figure 2.** Stirred tank fermenters containing immobilized-*Weissella cibaria* and different productive media including basal medium with soybean oil (A), basal medium with waste frying oil (B) and coconut water with waste frying oil (C).


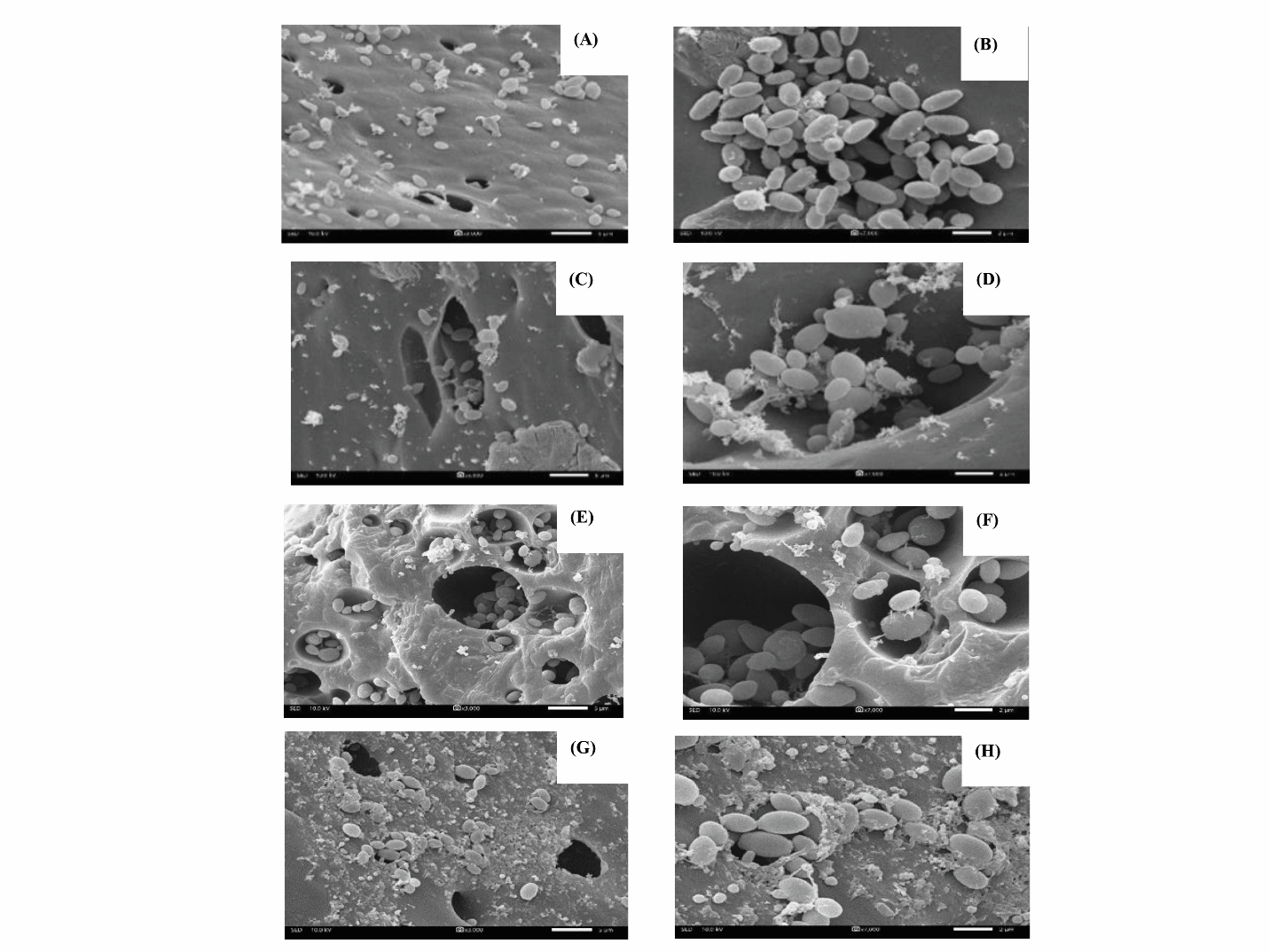


**Supplementary Figure 3.** SEM images of immobilized *Weissella cibaria* PN3 from a stirred tank fermenter containing basal medium with soybean oil after the 1^st^ (A and B) and 3^rd^ production cycles (C and D) and waste coconut water with waste frying oil after the 1^st^ (E and F) and 3^rd^ production cycles (G and H). The 3000x and 7000x magnified images are shown on the left and right, respectively. The immobilized cells from the 1^st^ production cycle were collected before the cell washing process, while those from the 3^rd^ production cycle were collected after the cell washing process.

**(F)**

**(E)**

**(B)**

**(A)**

**Supplementary Figure 4.** Determination of critical micelle concentrations of glycolipids from different productive media; crude extracellular (A) and cell-bound glycolipids (B) produced from basal medium with soybean oil; crude extracellular (C) and cell-bound glycolipids (D) produced from basal medium with waste frying oil and crude extracellular (E) and cell-bound glycolipids (F) produced from waste coconut water with waste frying oil.

**732 C-H**

**1461C=H**

**1268 C=H**

**2921 CH_2_CH_3_**

**2856**

**3279 OH**

**2933 CH_2_CH_3_**

**1632 C=O**

**(D)**

**(B)**

**(C)**

**(A)**

**Supplementary Figure 5.** FTIR spectra of purified extracellular (A) and cell-bound glycolipids (B) produced from basal medium with soybean oil; and purified extracellular (C) and cell-bound glycolipids (D) produced from waste coconut water with waste frying oil.

**3. Cost analysis of glycolipid and lipopeptide production process and the efficiency of biosurfactant-based dispersant from waste substrates**


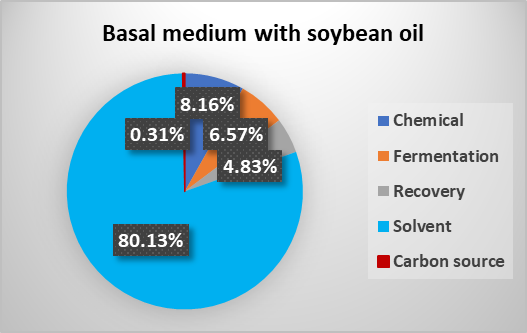


**(A)**


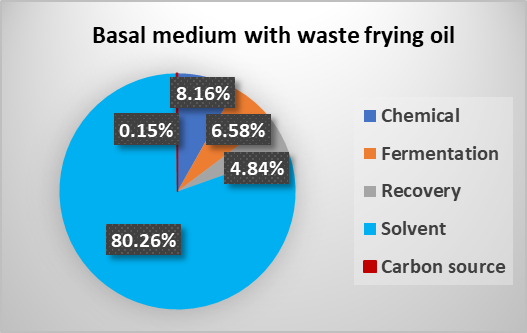


**(B)**


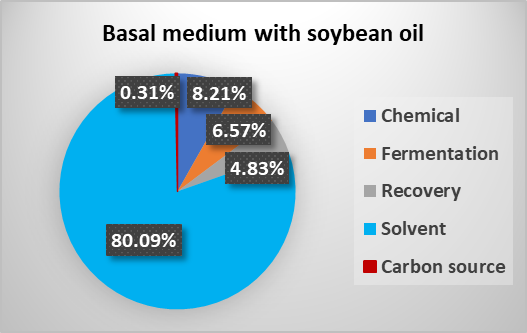


**(D)**


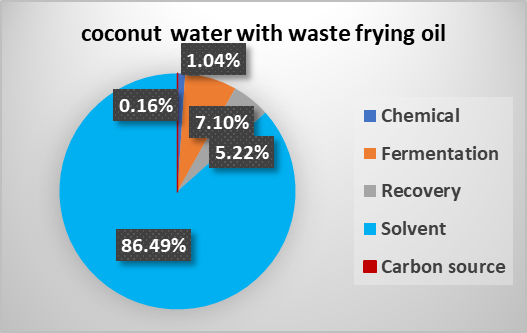


**(C)**


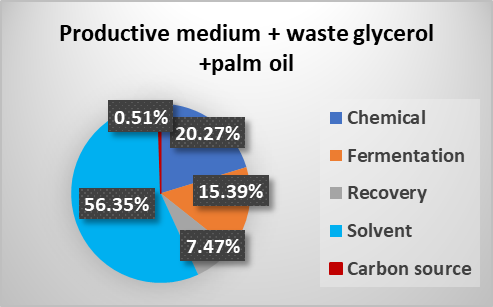


**(E)**

**Supplementary Figure 6.** Cost analysis of glycolipid production process using different productive media in stirred tank fermenter (A-C) and shaking flask (D); and lipopeptide production process (E).


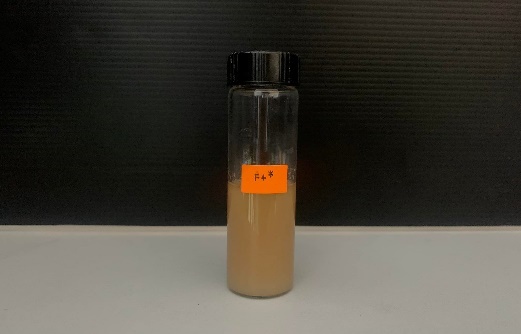


**(A)**

**(B)**

#### Supplementary Figure 7. Characteristic of F2* dispersant formulation (A) and its dispersion effectiveness with various oils (B). BKC and ARL/AXL were obtained from Thai Oil Public Company Limited. Fuel oil was obtained from Bangchak Corporation Public Company Limited and engine oil and gasoline were purchased from PTT Public Company Limited.
